# Millisecond-level transient heating and temperature monitoring technique for ultrasound-induced thermal strain imaging

**DOI:** 10.7150/thno.95997

**Published:** 2025-01-01

**Authors:** Mengyue Chen, Zhiyu Sheng, Ran Wei, Bohua Zhang, Howuk Kim, Huaiyu Wu, Yu Chu, Qiyang Chen, Andrew Breon, Sibo Li, Matthew B. Wielgat, Dhanansayan Shanmuganayagam, Edith Tzeng, Xuecang Geng, Kang Kim, Xiaoning Jiang

**Affiliations:** 1 Department of Mechanical and Aerospace Engineering, North Carolina State University, Raleigh, NC, USA.; 2 Department of Medicine, University of Pittsburgh, Pittsburgh, PA, USA.; 3 Department of Bioengineering, University of Pittsburgh, Pittsburgh, PA, USA.; 4 Blatek Inc., Boalsburg, PA, USA.; 5 Shenqi Medical (USA) Sirius Technologies Ltd., Boston, MA, USA.; 6 Department of Mechanical Engineering, Inha University, Incheon, South Korea.; 7 Department of Animal and Dairy Sciences, University of Wisconsin-Madison, Madison, WI, USA.; 8 Department of Surgery, University of Wisconsin School of Medicine and Public Health, Madison, WI, USA.; 9 Center for Biomedical Swine Research & Innovation, University of Wisconsin-Madison, Madison, WI, USA.; 10 Department of Surgery, University of Pittsburgh, Pittsburgh, PA, USA.

**Keywords:** vulnerable atherosclerotic plaque, theranostic ultrasound, ultrasound-induced thermal strain imaging, transient ultrasound-induced heating, transient temperature monitoring

## Abstract

**Background:** Ultrasound-induced thermal strain imaging (US-TSI) is a promising ultrasound imaging modality that has been demonstrated in preclinical studies to identify a lipid-rich necrotic core of an atherosclerotic plaque. However, human physiological motion, e.g., cardiac pulsation, poses challenges in implementing US-TSI applications, where achieving a millisecond-level temperature rise by delivering acoustic energy from a compact US-TSI probe is a key requirement. This study aims to develop a transient ultrasound heating and thermocouple monitoring technique at the millisecond level for US-TSI applications.

**Methods:** We designed, prototyped, and characterized a novel US-TSI probe that includes a high-power, 3.5 MHz heating transducer with symmetrical dual 1D concave array. Additionally, millisecond-level temperature monitoring was demonstrated with fast-response thermocouples in laser- and ultrasound- induced thermal tests. Subsequently, we demonstrated the prototyped US-TSI probe can produce a desired temperature rise in a millisecond-short time window *in vitro* phantom and *in vivo* animal tests.

**Results:** The prototyped US-TSI probe delivered zero-to-peak acoustic pressure up to 6.2 MPa with a 90 V_PP_ input voltage. Both laser- and ultrasound- induced thermal tests verified that the selected thermocouples can monitor temperature change within 50 ms. The fast-response thermocouple confirmed the transient heating ability of the US-TSI probe, achieving a 3.9 °C temperature rise after a 25 ms heating duration (50% duty cycle) in the gel phantom and a 2.0 °C temperature rise after a 50 ms heating duration (50% duty cycle) in a pig model.

**Conclusions:** We successfully demonstrated a millisecond-level transient heating and temperature monitoring technique utilizing the novel US-TSI probe and fast-response thermocouples. The reported transient ultrasound heating and thermocouple monitoring technique is promising for future *in vivo* human subject studies in US-TSI or other ultrasound-related thermal investigations.

## Introduction

Atherosclerosis remains one of the most complex cardiovascular diseases (CVDs) to diagnose, threatening numerous patients' lives 1-5. The process of atherosclerosis begins with intimal inflammation, necrosis, fibrosis, and calcification at specific artery sites, followed by plaque buildup in the inner lining of arteries, leading to the thickening of artery walls and reduction of blood flow 6-9. This lessens the oxygen and nutrients reaching the brain (carotid arteries), heart (coronary arteries), and other organs or tissues 10-12. Atherosclerotic plaques are classified as stable, with a thick fibrous cap and small lipid core, or vulnerable, with a thin fibrous cap and large lipid core. The vulnerable plaque is friable, and the rupture of plaques may diminish blood flow or completely occlude the arteries, leading to sudden cardiac death or acute ischemic stroke. Thus, early detection of subclinical atherosclerosis is crucial for effective prevention of sudden and fatal cardiovascular events 4,13. To identify vulnerable atherosclerotic plaque, various diagnostic modalities have been proposed, such as invasive and non-invasive approaches 4,14-16. Typical invasive plaque imaging modalities include intravascular magnetic resonance imaging (IVMRI), intracoronary optical coherence tomography (OCT), intracoronary near-infrared spectroscopy (NIRS), and intravascular ultrasound (IVUS), including the more recent advancement of super-harmonic IVUS 17-23. Such miniaturized, catheter-based, intravascular imaging modalities provide micrometer-level spatial resolution and offer the capability or potential to visualize plaques prone to rupture. However, these techniques necessitate surgery and have limited accessibility. Of the non-invasive imaging modalities, computed tomography (CT) suffers from limited (millimeter-level) spatial resolution, while magnetic resonance imaging (MRI) is hindered by restricted (centisecond-level) temporal resolution 24-27. Positron emission tomography (PET) is also effective for early detection of atherosclerosis by assessing molecular-level changes in tissue activity; however, it involves ionizing radiation and costly procedures 28,29.

Ultrasound has demonstrated efficiency and effectiveness in medical imaging, owing to its exceptional spatial and temporal resolution, non-ionizing nature, and affordability. Yet, traditional ultrasound imaging struggles to detect atherosclerotic plaques because the acoustic properties of the plaque closely resemble those of the surrounding tissue. However, the ultrasound-induced temperature rise within the tissue leads to differential changes in sound speed within lipid- and water-bearing tissues, allowing for the distinction of lipid-laden tissue from the surrounding tissue primarily composed of water. As vulnerable atherosclerotic plaques have higher proportions of lipids within the core, such plaques can be identified by 1) recording the ultrasound radiofrequency data before and after the induced temperature rise, 2) tracking the temperature rise-induced echo shifts change in time of flight, and 3) calculating the apparent strain (i.e., thermal strain or temporal strain) 30,31. This offers a new capability by utilizing ultrasound for the initial heating and subsequent imaging of vulnerable atherosclerotic plaque. Such innovative theranostic modality, merging therapeutic ultrasound with diagnostic ultrasound, is referred to as ultrasound-induced thermal strain imaging (US-TSI) 32,33.

**Figure 1:**
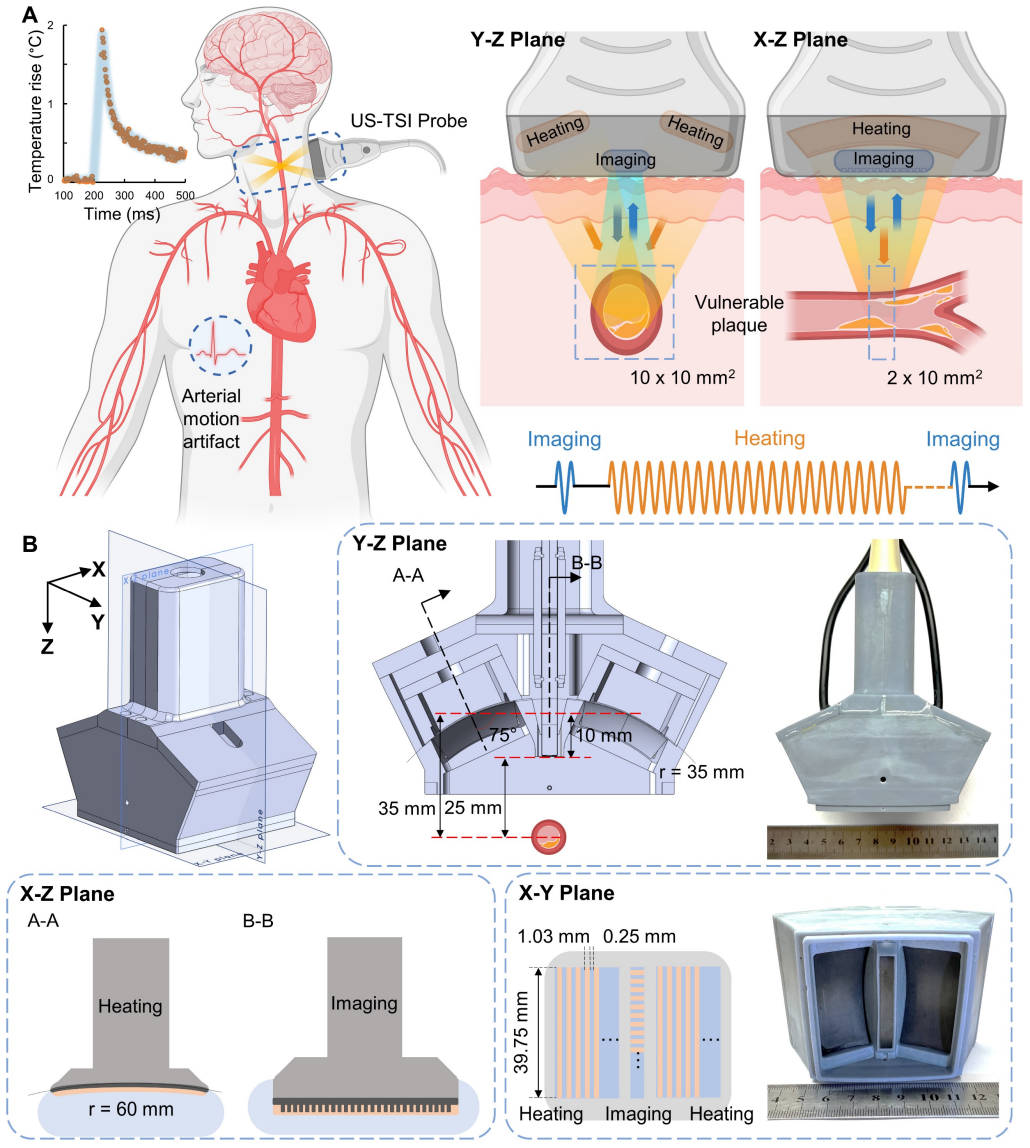
** Overview of US-TSI probe for vulnerable plaque detection. (A)** Sketch in the short- and long-axis direction for plaque detection using a US-TSI probe in the human artery, with blue arrows/cycles depicting the imaging process and orange arrows/cycles depicting the heating process. **(B)** Schematic and photo of prototyped US-TSI probe, including 3D model and 2D model in XZ, XY, and YZ planes. The figure was partially created by Biorender.com.

The development of US-TSI dates back a decade and a half when Huang *et al.* first demonstrated the feasibility of inducing and imaging thermal strain using a commercial ultrasound scanner in a gelatin phantom with a cylindrical rubber inclusion 30. Kim *et al.* further proved the potential of US-TSI for detecting and monitoring atherosclerotic plaque using rabbit kidneys *ex vivo* 34. The first *in vivo* animal (rabbit) studies by Mahmoud *et al.* confirmed that US-TSI can monitor lipids in atherosclerotic plaque 31. Besides these studies, work has been done to enhance the performance of US-TSI devices by developing high-powered US-TSI systems to increase heating speed, employing advanced ultrasound beamforming algorithms to enlarge heating volume, or implementing compensation algorithms to mitigate physiological motion artifacts. For example, Stephens *et al.* developed a novel US-TSI probe consisting of six 3.5 MHz heating transducers, which can produce a 3 °C temperature in 2 s 35. Nguyen *et al.* and Khalid *et al.* employed a multi-focus beamforming algorithm on a linear array and a curved linear array, respectively, to achieve a relatively large and uniform heating area within a few seconds 36,37. Yin *et al.* proposed and refined a compensation method to prevent artifacts caused by respiration within seconds 38,39. In summary, current US-TSI devices, particularly concerning their heating performance (Table S1), are better suited for mitigating physiological motion artifacts such as those caused by respiration rather than cardiac pulsation. Further improvements are necessary to optimize US-TSI devices for clinical applications where physiological motion (e.g., cardiac pulsation) exists. The displacement of arterial wall motion within a 1/8th cardiac cycle, lasting 75 - 125 ms, is generally less than 1 mm and thus practical for plaque imaging 40. Additionally, major human arteries, like the carotid, are typically 4 - 7 mm in diameter 41. This necessitates generating a 10 × 10 mm^2^ heating area to cover the artery adequately. Thus, the desired US-TSI device must heat a 2 × 10 × 10 mm^3^ volume and produce an approximately 2 °C increase within 50 ms (Figure 1) 42.

After the desired US-TSI prototype is developed, it's essential to monitor transient temperature changes within biological tissue to validate its rapid heating performance. Infrared (IR) thermometry, magnetic resonance (MR) thermometry, and thermocouple thermometry are mainly used for biological temperature monitoring. However, IR thermometry utilizes the emitted infrared radiation from objects to assess temperature variations, limiting its capabilities to measure surface temperatures 43. MR thermometry exploits the proton resonance frequency shift to evaluate the temperature change deep inside the body; however, it lacks the capability of transient temperature monitoring due to second-level temporal resolution 44,45. Thermocouple thermometry relies on the Seebeck effect, where a potential is generated when two dissimilar metals in contact are exposed to temperature change 46. These attributes of simplicity, versatility, and durability have contributed to the widespread adoption of thermocouples in various temperature monitoring applications. Importantly, thermocouples exhibit rapid response times, reaching temporal resolutions on the order of milliseconds 47. This quality demonstrates their potential for validating the transient heating performance of US-TSI devices.

The principal objective of this study is to develop techniques for millisecond-level transient ultrasound heating and thermocouple monitoring tailored for US-TSI applications. A novel US-TSI probe equipped with a high-power heating transducer was designed, simulated, fabricated, and characterized. The feasibility of transient temperature monitoring approaches was demonstrated using commercial ultra-fast response thermocouples in experiments involving laser- and ultrasound- induced heating. Finally, we conducted *in vitro* phantom and *in vivo* animal tests to showcase the viability of inducing transient heating with the prototyped US-TSI probe.

## Materials and Methods

### US-TSI Probe System

The US-TSI probe aims to provide satisfactory heating capabilities in speed and volume and image the human carotid artery with sufficient spatial and temporal resolution. As illustrated in Figure 1, the probe is mainly comprised of an imaging transducer (blue part) and a heating transducer (orange part). The imaging transducer is a 1D linear array consisting of 192 elements, whereas the heating transducer is a symmetrical dual 1D concave array consisting of 16 elements for each side. The imaging transducer and heating transducer are linked to a Verasonics ultrasound system (Vantage 256™, Verasonics Inc., WA, USA) with an external power supply for pulse sequence control. The external power supply (QPX600DP, Aim and Thurlby Thandar Instruments, Ltd., UK) supports a maximum input voltage of 190 V_PP_, an average power of up to 1200 W, and a maximum current of 2.0 A per channel for pulse lengths lasting up to several seconds. To elevate the temperature in the region of interest, the heating transducer delivers a pulse train with a duty cycle of 50 % that overall lasts less than 50 ms, and there are 240 cycles in each pulse. The pulse from the imaging transducer has only 2 cycles for each transmit and it captures the echoes both before and after the heating sequences. The driving voltage is kept constant to avoid repeatedly charging and discharging of the hardware capacitor during short periods. In this way, the imaging and heating transducer have to share the same driving voltage that is much higher than needed for the imaging array. Therefore, a programmable apodization was applied to the imaging array to scale down the transmit power. Based on our previous studies, the center frequency of the heating transducer is set at 3.5 MHz to strike a balance between the required ultrasound penetration depth and the desired acoustic energy absorption 35. We also applied the dual focal depth method on the heating transducer to enlarge the ultrasound beamwidth. The lateral focal depth was set at 40 mm (YZ-plane) based on phase delay, while the elevational focal depth was set at 60 mm (XZ-plane) based on curvature radius 42. Other design specifications are summarized in Table 1.

### Heating Transducer Design and Simulation

The primary innovation of the US-TSI probe lies in the development of a heating transducer exhibiting superior temperature elevation performance. In our previous study, we preliminarily designed the heating transducer by simulating the ultrasound beam pattern and temperature response 42. The simulated results demonstrated that the designed heating transducer generally meets the heating volume and speed requirements. We further developed the heating transducer in the present study by determining the acoustic lens and phase delay.

Figure 1B depicts the design layout of the US-TSI probe, where the symmetrical dual heating arrays are situated 30 mm apart on both sides of the imaging array. Each heating array rotates 25° to target the location 25 mm below the imaging array. Furthermore, compared to the previous design, we chose PZT-5A instead of PZT-4 as active materials and selected dual 1D arrays instead of dual 1.5D arrays as the transducer configuration 42. This new design enlarged the size of each heating array element and electrically connected the five sections in one channel to achieve a reduced electrical impedance approaching 50 Ω. The soft piezoelectric material, PZT-5A, was selected as the active material for heating array elements owing to its high piezoelectric constants and mechanical strength, with Al_2_O_3_/epoxy and air as the materials for the matching and backing layers, respectively. A concave acoustic lens, constructed from graphite and having a radius of 41 mm, was also attached to the surface of the heating transducer to avoid side lobes. To obtain a larger volume for the heating zone, the multi-focus beamforming technique was implemented on the heating transducer by programming phase delays on each heating element 36.

The ultrasound beam pattern of the designed heating transducer, especially with an acoustic lens and phase-delay algorithm, was optimized and confirmed using finite element simulation to make sure the focal area can cover human carotids with a diameter of 4 - 7 m. Due to the massive computational cost, we only simulated acoustic pressure distribution using COMSOL Multiphysics (6.1, COMSOL Inc., Stockholm, Sweden) in a 2D plane. The acoustic-piezoelectric model is illustrated in Figure S1, and the corresponding simulation parameters are summarized in Table S2 48-50. To determine the phase delay on each element, we followed our previous study, which showed that the delay profile for a linear array transducer generally exhibits a parabolic shape with two distinct notches or dips near the peak 36. To apply phase delay in the finite element simulation, we first calculated the time delay between each element, then converted the time delay to phase delay, and finally applied the input voltage of each element using a complex-valued number (Figure S1). By checking the simulated ultrasound beam pattern, we selected the appropriate phase delays of each element, illustrated in Figure S1.

### Heating Transducer Fabrication and Characterization

We first prototyped three single heating transducer elements with three materials: PZT-5A, PZT-5H, and PMN-PT. After conducting characterization consisting of electrical impedance, pulse-echo, and hydrophone tests, we adopted PZT 5A as the active layer due to its superior loop and transmitting sensitivities. Detailed comparisons are summarized in Table S3. The matching layer was fabricated by mixing Al_2_O_3_ powder (ALR-1005-01, Pace Technologies Inc., AZ, USA) and epoxy resin (EPO-TEK 301, Epoxy Technology, Inc., MA, USA), while the backing layer was prepared using the mixture of air-bubble powder (Blatek Industries, Inc., PA, USA) and epoxy resin (EPO-TEK 301, Epoxy Technology, Inc., MA, USA). Then, a 10-μm thick Parylene-C layer was coated to encapsulate the heating transducer for waterproofing. Finally, we integrated the heating and imaging transducer into a 3D printing housing according to the design layout to prototype the US-TSI probe.

**Figure 2:**
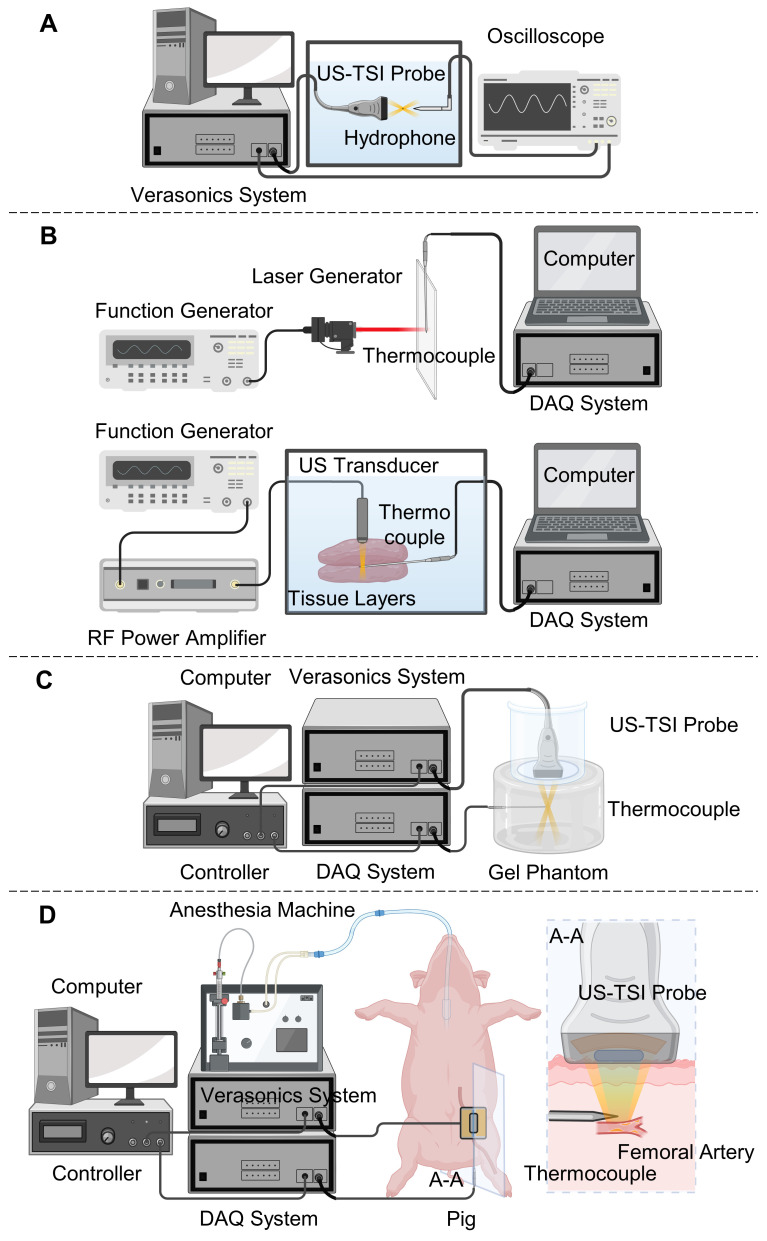
** Sketches of experiment setup** for **(A)** hydrophone tests to characterize the US-TSI probe; **(B)** laser-induced and ultrasound-induced thermal tests to check the performance of the fast response thermocouples for transient temperature monitoring, **(C)** *in vitro* phantom tests, and **(D)** *in vivo* animal tests to check the transient heating capability of the prototyped US-TSI probe. The figure was created with Biorender.com.

After the fabrication of the US-TSI probe, we measured the electrical characteristics of each heating transducer element, including the impedance and phase spectrum, by a precision impedance analyzer (4294A, Agilent Technologies Inc., CA, USA). The output and distribution of its acoustic pressure were measured using a hydrophone to assess the acoustic characteristics of the prototyped US-TSI probe. As illustrated in Figure 2A, a water tank filled with degassed water was utilized to accommodate both the US-TSI probe and the hydrophone. Verasonics ultrasound system was used to control the US-TSI probe. Specifically, a sinusoidal pulse of 10 cycles per 1 ms was transmitted to drive the heating transducer. A calibrated bullet hydrophone (HGL-0085, ONDA Corporation, CA, USA) connected with a 20 dB preamplifier (AH-2020, ONDA Corporation, CA, US) was placed in the region of interest behind the heating transducer. Given that the bullet hydrophone could not measure high-pressure/intensity ultrasound, we operated the heating transducer with input voltages ranging from 0 to 20 V_PP_. The measurement was repeated three times for each input voltage to obtain averaged acoustic outputs. The acoustic pressure field was acquired and recorded using a digital oscilloscope (DSO7104B, Agilent Technologies, Inc., CA, USA) with the assistance of the 3-axis positioning system. Specifically, we conducted scans of the acoustic pressure distribution in the XZ and YZ planes, employing a step size of 0.5 mm in the X and Y directions and 1.0 mm in the Z direction. In addition, we measured zoom-in acoustic pressure fields along the X and Y directions with a step size of 0.1 mm.

### Thermocouple Preparation and Verification

We opted for commercially available thermocouples with milliseconds response time to capture the transient temperature change. In selecting thermocouple types, we chose Type T (copper-constantan) due to its exceptional accuracies (approximately 1.0 °C) and ample temperature ranges (-250 to 350 °C) compared to Type J, K, and E. To record the signal from the thermocouple, we connected a data acquisition (DAQ) module (OM-DAQ-USB-2401, Omega Engineering, Inc., CT, USA) to the thermocouple, operating at a sampling rate of 500 Hz. Other details of two selected thermocouples utilized in this study are summarized in Table S4.

To verify the capability of selected thermocouples, we need robust and stable heating sources known to deliver a comparable amount of energy and produce a similar temperature to the new US-TSI probe. In this paper, we used a laser line generator to perform the heating tests in the air and a single-element ultrasound transducer to perform the heating tests in an excised porcine tissue. As depicted in Figure 2B, a laser line generator driven by a function generator (33250A, Agilent Technologies, Inc., CA, USA) produced a laser line lasting 50 ms per 2 s. The laser line generator was mounted on a 3-axis motion stage to ensure effective capture of the laser beam by the tip of the thermocouple. The resultant electrical voltage change was recorded by the DAQ system and then translated into a temperature change by the computer.

Regarding the ultrasound-induced thermal tests (see Figure 2B), a single-element ultrasound transducer (Blatek Industries, Inc., PA, USA) was driven by a function generator and an RF power amplifier (75A250A, Amplifier Research Corporation, PA, USA), and generated focused ultrasound waves lasting 50 ms per 2 s. The frequency, acoustic pressure, and focal depth of generated ultrasound waves are 950 kHz, 2.4 MPa, and 20 mm, respectively. A water tank filled with degassed water at 35 °C was employed to house the ultrasound transducer, the thermocouple, and the layers of the porcine tissue. We positioned the thermocouple between two 20 mm-thick porcine tissue layers and mounted the ultrasound transducer on a 3-axis motion stage to ensure effective capture of the ultrasound beam by the tip of the thermocouple.

### *In Vitro* Phantom Tests

Gel phantoms with a gelatin concentration of 5% (w/v gelatin powder/saline) were prepared to mimic biological tissue. 25 g gelatin powder (G2500, MilliporeSigma Corporation, MA, USA) was first added to 500 mL phosphate-buffered saline (PBS) solution (Gibco, Thermo Fisher Scientific Inc., MA, USA) at 49 °C using a stirring hot plate (PC420D, Corning Inc., NY, USA). Afterward, we carefully removed any trapped air bubbles within the solution and stirred it thoroughly with a magnetic stirrer until the gelatin was dissolved completely. The solution was then cooled to 38 °C and mixed with 5 g of cellulose powder (S3504, MilliporeSigma Corporation, MA, USA). We subsequently stirred the mixture until fully dissolved and cooled it to 18-21 °C. Finally, the solution was poured into a container and placed in a refrigerator for solidification.

Following the preparation of the gel phantom, we proceeded to perform ultrasound-induced thermal tests using the prototyped US-TSI probe, aided by a fast-response thermocouple for monitoring transient temperature changes. As illustrated in Figure 2C, the US-TSI probe was submerged in a plastic container filled with degassed water and subsequently positioned on the top surface of the gel phantom. This was done to easily eliminate any air bubbles trapped or generated between the US-TSI probe and the gel phantom, as opposed to using ultrasonic couplant gel. In addition, a thin layer of plastic wrap was used to replace the bottom of the plastic container, ensuring adequate transmission of acoustic energy. We inserted the fast-response thermocouple into the region of interest of the gel phantom, which is about 35 mm below the heating transducer and 25 mm below the imaging transducer. Furthermore, a controller linked to a computer was employed to synchronize the Verasonics system and the DAQ system. The Verasonics ultrasound system operated the heating transducer with different input voltages, ranging from 40 V_PP_ to 90 V_PP_, for a duration of 25 ms. The DAQ system recorded the electrical voltage change of the fast-response thermocouple at a sampling rate of 500 Hz. It should be noted that each condition was repeated three times, and the results were averaged to ensure reliable temperature monitoring.

### *In Vivo* Animal Tests

The animal test was performed on a Wisconsin Miniature Swine™ (WMS™) to validate the effectiveness of the US-TSI probe-induced temperature increase *in vivo*. Under a protocol approved by the Institutional Animal Care and Use Committee (IACUC) at the University of Pittsburgh, the pig was anesthetized and underwent ultrasound imaging and local heating using the prototyped US-TSI probe. As illustrated in Figure 2D, we inserted the fast-response thermocouple under the guidance of ultrasound imaging. Considering the future US-TSI applications on the human carotid artery at a similar depth to a pig femoral artery under the skin, we targeted a homogenous muscular area near the femoral artery of the left leg. The US-TSI probe, filled with ultrasonic couplant gel and covered using a very thin plastic wrap (Figure S2), rested on the pig leg using a probe holder. We adjusted the position of the US-TSI probe and used visual feedback from ultrasound imaging to ensure the precise positioning of the thermocouple. To demonstrate and compare the effectiveness of the acoustic heating, we executed the acoustical heating sequence using the Verasonics ultrasound system with an external power supply as used in the previously mentioned phantom tests and recorded temperature curves when the tip of the thermocouple was identified at three different locations (0, 2, 5 mm off focus). The temperature data was stored using the DAQ system. The Verasonics ultrasound system operated the heating transducer with an input voltage of 90 V_PP_, a pulse cycle of 240, a duty cycle of 50%, and a heating duration of 50 ms. The DAQ system recorded the electrical voltage change of the fast-response thermocouple at a sampling rate of 500 Hz.

## Results

### Heating Transducer Development

Figure S3 illustrates the simulated acoustic pressure fields of the heating transducer in the YZ plane, which consisted of various conditions, including the presence and absence of acoustic phase delay and acoustic lens. All the simulated acoustic pressure fields were normalized to the peak pressure of each ultrasound beam. It was observed that using both the acoustic lens and phase delay is essential to prevent wide, unfocused beam patterns (Figure S3A), low acoustic pressure (< -20 dB) between focal points (Figure S3B), and a focal area shift above the region of interest (Figure S3C). Additionally, we selected the phase delay illustrated in Figure S1 to get an approximately 8 x 16 mm^2^ focal area (-12 dB) illustrated in Figure S3D and Figure S4, which fully cover human carotids with a diameter of 4 - 7 mm. It was also noted that when the axial distance is less than 13 mm, the sound pressure level (SPL) drops below -30 dB (Figure S3D). Therefore, we opted to position the imaging array at an axial distance of 10 mm. Using the optimized design verified by simulated results, the US-TSI probe was prototyped accordingly (refer to Figure 1).

### Heating Transducer Characterization

**Figure 3:**
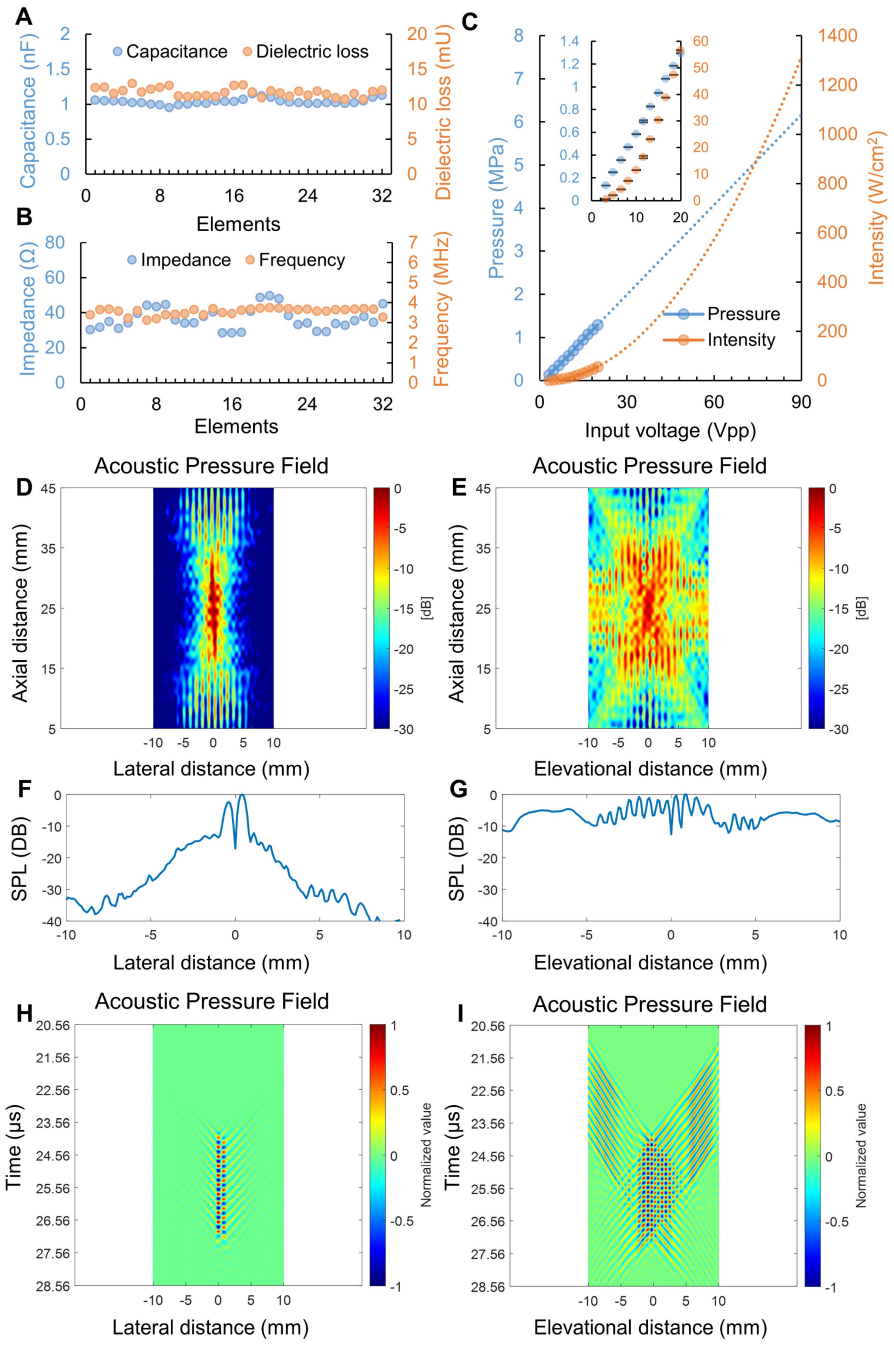
** Measured heating transducer characterization results. (A)** capacitance and dielectric loss of each heating transducer element at 1 kHz; **(B)** electrical impedance at 3.5 MHz and minimum impedance frequency of each heating transducer element; **(C)** acoustic pressure and intensity output of the heating transducer (n = 3); acoustic pressure filed of the heating transducer in the **(D)** XZ plane and **(E)** YZ plane; zoom-in acoustic pressure distribution along the **(F)** X- and **(G)** Y-directions; temporal acoustic pressure filed of the heating transducer at focal depth along the **(H)** X- and **(I)** Y-directions.

**Figure 4:**
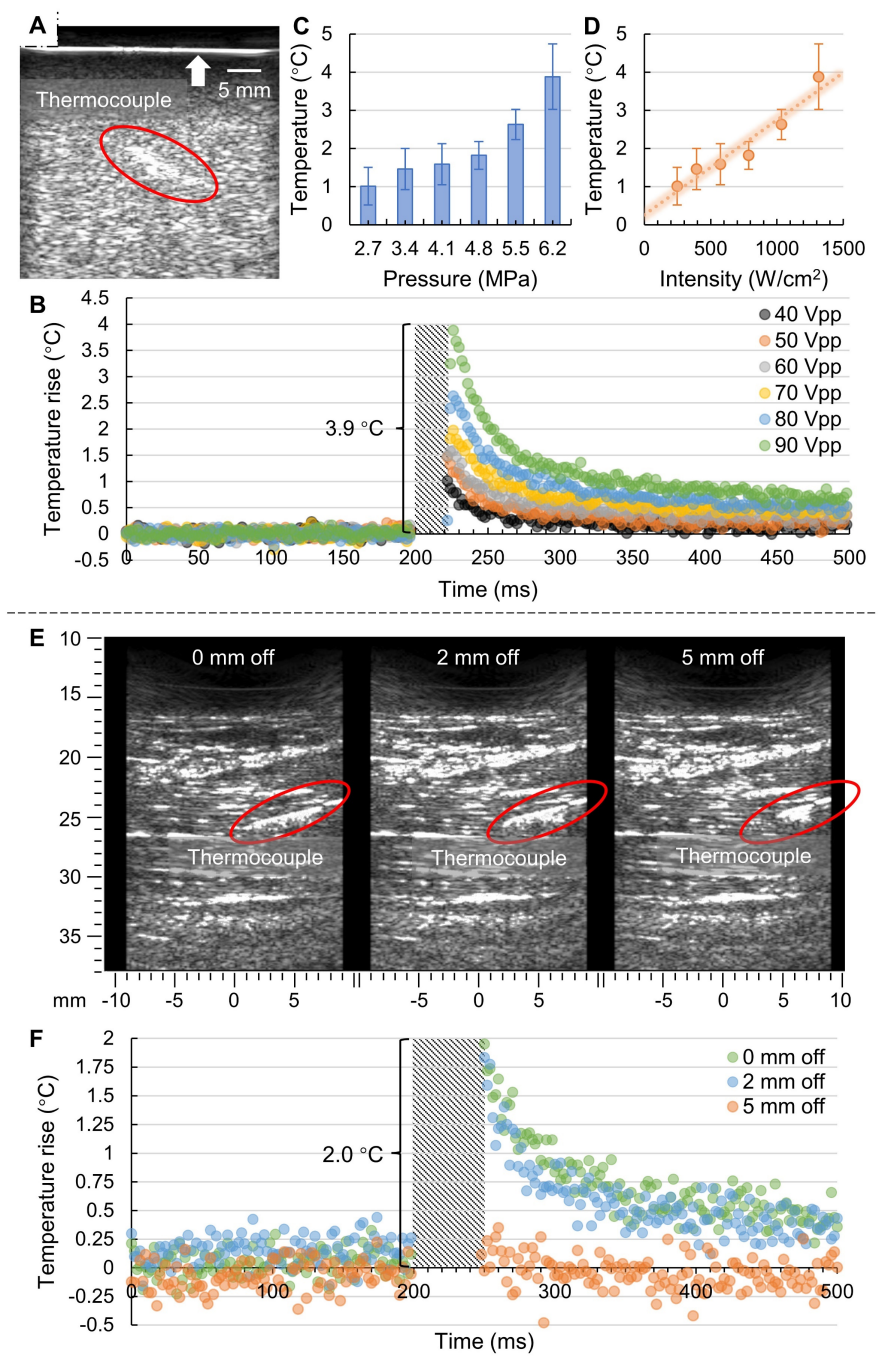
** Results of *in vitro* phantom tests and *in vivo* animal tests. (A)** B-mode imaging in phantom from the imaging transducer; **(B)** measured transient temperature curve in phantom for various input voltages (n = 3); **(C)** the relationship between temperature rise and acoustic pressure; **(D)** the relationship between temperature rise and acoustic intensity, where the error bar represents the standard deviation from three repeated measurements (n = 3); **(E)** B-mode imaging in pig leg shows thermocouple locations; and **(F)** measured transient temperature curve in pig leg for three different thermocouple locations (n = 1).

Figure 3 illustrates the electrical and acoustic characteristics of the prototyped heating transducer. The capacitance and dielectric loss at 1 kHz for the 32 heating transducer elements were 1.04 ± 0.04 nF and 11.71 ± 0.61 mU, respectively. Meanwhile, the electrical impedance at operating frequency (i.e., 3.5 MHz) and minimum impedance frequency for the 32 heating transducer elements were 36.65 ± 6.14 Ω and 3.55 ± 0.17 MHz. The detailed electrical characteristics of each heating transducer element are displayed in Figure 3A&B and Table S5. In Figure 3C, the zero-to-peak acoustic pressure and spatial-peak pulse-average acoustic intensity (I_SPPA_) were depicted in blue and orange colors, respectively. We extrapolated acoustic pressure and intensity by applying linear and 2^nd^-order polynomial trendlines, respectively. The result indicated that an input voltage of 90 V_PP_ applied to the heating transducer could generate approximately a peak pressure of 6.2 MPa and an I_SPPA_ of 1300 W/cm^2^. Figures 4D and 4E display the measured acoustic pressure fields of the heating transducer in the XZ and YZ planes. It should be noted that the axial distance in Figures 4D and 4E refers to the distance between the hydrophone and the imaging transducer. Spline interpolation was employed to enhance the smoothness of the map depicting the normalized acoustic pressure field. The measured results in Figures 4D and 4E show that the focal depth of the heating transducer was at the axial distance of 25 mm, aligning with the design objectives. The -12 dB beamwidth was measured to be about 2 mm in the X-direction and 10 mm in the Y-direction. It is clear that an ultrasound beam pattern resembling an “X” shape, as shown in Figure 4E, was formed due to the configuration of symmetrical dual heating arrays. Figures 4F and 4G present the zoomed-in acoustic pressure distribution, expressed as SPL, in the X- and Y-directions at a focal depth of 25 mm. It is interesting to observe multiple crests and troughs occurring in the Y-direction. In addition, as illustrated in Figures 4H and 4I, we plotted the spatial acoustic pressure distribution (at a focal depth of 25 mm) along the X-axis and the temporal acoustic pressure distribution (including a time window of 10-cycle tone burst) along the Y-axis. These temporal-spatial acoustic pressure distributions for both the lateral and elevational directions displayed the acoustic wave propagation and interference from the dual ultrasound heating transducer array.

### Thermocouple Verification

Figure S5 illustrates the representative temperature curves in both laser-induced and ultrasound-induced thermal tests. It can be observed, from the raw data dots, that the variation in temperature changes is approximately 0.7 °C in the air and 0.9 °C in the tissue. Thus, a moving average (over 10 periods) was applied during the signal processing to mitigate noise effects and measurement variations. From the moving average line, it is noticeable that a temperature peak occurs every 2 s, with each temperature rise lasting about 50 ms for each peak. This is in accordance with the set period of laser/ultrasound pulse width and pulse repetition period. This result indicated that the selected thermocouples can be used to monitor millisecond-level trainset temperature.

### *In Vitro* Phantom Tests

We performed in vitro phantom tests using the prototyped US-TSI probe after heating transducer characterization and thermocouple verification. The B-mode image captured by the imaging transducer is presented in Figure 4A, wherein the thermocouple is marked by a circle. Notably, the bright line at the top (indicated by an arrow) represents the boundary between the gel phantom with scatterers and the water contained by a layer of plastic wrap. Figure 4B illustrates the transient temperature changes during a 500 ms period in a plot of normalized temperature over time. It is important to note that due to the strong electromagnetic interference from the prototyped US-TSI probe, we removed the corresponding error data and marked the affected period using a diagonal stripe pattern. It can be observed that applying an input voltage of 90 V_PP_ on the heating transducer can cause a temperature rise of 3.9 °C after a 25 ms heating duration. The corresponding acoustic pressure and intensity at the heating focal zone were approximately estimated as 6.2 MPa and 1300 W/cm^2^, respectively, based on the heating transducer sensitivities depicted in Figure 3C. While the detailed temperature curve within the 25 ms heating duration cannot be observed due to intense electromagnetic interference, it is evident that higher input voltage contributed to higher transient temperature rise. We then plotted the relationship between normalized temperature, acoustic pressure, and intensity, as shown in Figure 4C and Figure 4D. The error bars for the temperature rise correspond to the standard deviations across three repeated thermocouple measurements. There appears to be a linear dependence between the temperature rise and the acoustic intensity. The deviation between the measured data dots and estimated trend line might be attributed to (i) estimation error in predicting acoustic intensity in gel phantom rather than water and (ii) measurement error in placing the thermocouple in the focal zone of the ultrasound heating beam.

### *In Vivo* Animal Tests

The animal test results are presented in Figure 4. Figure 4E illustrates the B-mode images captured by the imaging transducer for three different thermocouple locations, wherein the thermocouple is marked by a red circle. The three thermocouple locations included one "at focus" case, positioned at the focal spot of the heating beam, and two "off focus" cases, positioned at 2 mm and 5 mm laterally off the focus. Figure 4F illustrates the corresponding transient temperature changes for the three thermocouple locations before and after the ultrasound heating. Like the previous phantom test results, the real temperature curve within the 50 ms heating duration cannot be monitored due to intense electromagnetic interference from the US-TSI probe. However, it is noteworthy that the temperature can rise roughly 2 °C after a 50 ms heating duration for both “0 mm off” (i.e., at focus) and “2 mm off”, indicating that the effective heating zone is larger than 4 mm in the lateral direction of the imaging transducer. For the thermocouple location of “5 mm off”, it is hard to observe any obvious temperature changes, indicating that the heating beam does not heat surrounding tissue outside the heating region of interest. Considering that the design requirement for the heating area size in the lateral direction of the imaging transducer is within 2 mm, these results demonstrate that the prototyped US-TSI probe delivers sufficiently high power for transient heating uniformly only within the target area, reflecting the effectiveness and safety for *in vivo* applications.

## Discussion

This study sought to develop a millisecond-level transient ultrasound heating and thermocouple monitoring technique for US-TSI applications. We first designed, prototyped, and characterized a novel US-TSI probe that included a heating transducer with exceptional heating capabilities. The simulation results, in terms of the acoustic pressure field in the YZ plane (Figure S3), emphasize the importance of applying an acoustic lens and a multi-focus beamforming algorithm. These measures can enlarge and homogenize the focal zone of the ultrasound heating beam. However, it should be acknowledged that the absence of 3D finite element simulation, attributable to substantial computational costs, posed challenges in the design of the heating transducer. Consequently, we opted for a compromise by (i) initially conducting a 3D acoustic simulation using Field II to validate the design specifications of the heating transducer in the previous study 42 and (ii) subsequently performing a 2D acoustic simulation using COMSOL to confirm the layout and phase delay of the heating transducer in the current study.

Regarding the acoustic characterization of the heating transducer, the measured transducer sensitivity aided us in estimating that the acoustic pressure of the prototyped US-TSI probe could reach 6.2 MPa (Figure 3C) when applying a 90 V_PP_ input voltage to the heating transducer. The measured pressure indicated the capability of delivering sufficient energy quickly. Ultimately when applying US-TSI in the clinical setting, this driving voltage needs to be scaled down to satisfy Food and Drug Administration (FDA) safety limits 51. For the acoustic pressure field shown in Figure 3E, the multi-focus beamforming and acoustic lens contributed to a wider beamwidth in the Y-direction compared to the X-direction. It is promising to see that the -12 dB beamwidth is roughly 10 mm, which is capable of heating major human arteries. Also, the multiple crests and troughs in both the X- and Y-directions are believed to be induced by ultrasound wave interference from the dual heating arrays (Figures 3D&E and Figure S4). The measured distance between the two neighboring crests/troughs is about 0.6 mm, which is closely aligned with the calculated value according to the fringe spacing equation for two-plane wave interference: 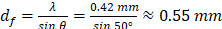, where 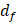 is known as the fringe spacing, 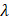 is the wavelength, and 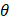 is the wave interference angle. In addition, considering that the conventional evaluation method for acoustic pressure field only accounts for the maximum ultrasound pressure in the spatial domain, we also involve the temporal acoustic pressure information to better observe the ultrasound wave propagation from the heating transducer. The normalized acoustic pressure field illustrated in Figures 3H and 3I reveal the interference patterns of ultrasound waveforms from the dual heating arrays, highlighting how the acoustic pressure varies with time and space. This new spatial-temporal evaluation metric might be promising for the acoustic characteristics of therapeutic ultrasound transducers in the future.

Apart from prototyping and characterizing the heating transducer, the approach for milliseconds-level temperature monitoring was also successfully demonstrated (Figure S5). This approach holds promise for future biological-related thermal studies such as TSI-based tissue characterization, TSI-based temperature monitoring, and ultrasound-induced thermal ablation 32,33,52. Nevertheless, it should be acknowledged that thermocouple thermometry has its drawbacks for ultrasound-induced thermal studies. The power cable of the ultrasound transducer needs to be carefully shielded and grounded to prevent the potential capacitive coupling effects. If not, the induced electromagnetic interference might disturb the thermocouple and induce “pick-up” errors 53. Moreover, evaluation of the impact of viscous heating artifacts on thermocouples is challenging, particularly for millisecond-level temperature monitoring 54. Addressing this issue will require further research and effort in the future.

Finally, utilizing the fast-response thermocouple, we successfully demonstrated the ability of the prototyped US-TSI probe to induce millisecond-level temperature changes in both a tissue-mimicking gel phantom and a muscular region near the femoral artery of a pig. In the *in vitro* tests, the heating transducer increased the temperature by 3.9 °C after a 25 ms sonication duration, suggesting a satisfactory heating capability and the margin for tuning *in vivo* to meet both heating and safety requirements. The *in vivo* tests later showed that the heating transducer enabled a 2.0 °C increase after a 50 ms sonication duration. The lower temperature rise with a longer heating duration might be due to an energy loss caused by blood perfusion under *in vivo* conditions 55. We further compared the heating capability of our prototyped US-TSI probe with that of current US-TSI devices, as summarized in Table S1. It can be observed that, with successful *in vitro* and *in vivo* demonstrations, our device advances the transient ultrasound heating capability from second-level to millisecond-level durations, which is promising for mitigating physiological motion artifacts like cardiac pulsation. In addition to heating durations, the performance of our US-TSI probe is also promising in terms of heating volume. A 4 mm effective heating width was observed in the lateral direction of the US-TSI probe (see Figures 4E&F), which is larger than the 2 mm -12 dB beamwidth of the ultrasound (see Figures 3D&F). This raises an interesting question: how can we determine the effective heating width based on the ultrasound beamwidth for millisecond-level duration? Meanwhile, since we could not precisely determine the thermocouple location in the evaluation direction of the US-TSI probe using ultrasound imaging *in vivo*, the effective heating area *in vivo* in that direction remains unknown. However, we expect it to be larger than 10 mm according to the -12 dB ultrasound beamwidth from both numerical simulation and hydrophone measurements. Overall, we remain optimistic about the potential of this novel US-TSI probe for future studies and applications in the field of US-TSI.

The next phases of the project will involve (i) conducting a benchtop investigation using gel phantoms to assess three crucial variables for TSI, namely thermal strain, temperature increase, and lipid concentration; (ii) undertaking an *in-vivo* study involving clinically relevant hyperlipidemic pigs to evaluate the performance and safety of the US-TSI probe and optimize the US-TSI protocol for clinical translation; (iii) initiating a pilot human study involving patients with atherosclerosis to demonstrate the capability of US-TSI in detecting plaque in the human carotid artery.

## Conclusions

To the best of our knowledge, this study marks the first successful demonstration of a millisecond-level transient heating and temperature monitoring technique in US-TSI. In this study, a novel US-TSI probe was developed, showcasing remarkable heating capabilities. The performance of the heating transducer was assessed by examining its acoustic pressure output and distribution. Additionally, an approach for transient temperature monitoring using fast-response thermocouples was established. Following that, we demonstrated that the prototyped US-TSI probe can induce millisecond-level transient heating. It is believed that the developed millisecond-level transient ultrasound heating and thermocouple monitoring technique is promising for future studies in the US-TSI field or other ultrasound-related thermal effects investigations.

## Supplementary Material

Supplementary figures and tables.

## Figures and Tables

**Table 1:** Design specifications for the US-TSI probe

Imaging Transducer		Heating Transducer	
Configuration	1D Linear Array	Configuration	Dual 1D Concave Arrays
Elements number	192	Elements number	2 x 16
Element pitch (X-direction^*^)	0.2 mm	Element pitch (Y-direction^*^)	1.03 mm
Element kerf (X-direction^*^)	0.05 mm	Element kerf (Y-direction^*^)	0.25 mm
Element height (Y-direction^*^)	4 mm	Element height (X-direction^*^)	39.75 mm
Center frequency	10 MHz	Center frequency	3.5 MHz
Focal depth	25 mm	Focal depth	60 mm (XZ plane) & 40 mm (YZ plane)
Material	PZT-5H	Material	PZT-5A

^*^: The lateral and elevation directions of the imaging array correspond to the X- and Y-directions, while for the heating array, the lateral and elevation directions are reversed, corresponding to the Y- and X-directions (Figure 1B).
