# Supplementary material for: Millisecond-level transient heating and temperature monitoring technique for ultrasound-induced thermal strain imaging
Source: Theranostics. 2025 Jan 1;15(3):815–27. doi: 10.7150/thno.95997 (PMC11700873; doi:10.7150/thno.95997)
Supplement: Supplementary file 1 — Supplementary figures and tables. [file thnov15p0815s1.pdf]

Supplementary Information

Millisecond-level transient heating and temperature monitoring technique for ultrasound-induced thermal strain imaging

Mengyue Chen[1, *], Zhiyu Sheng[2, *], Ran Wei[3], Bohua Zhang[1, 5], Howuk Kim[1, 6], Huaiyu Wu[1], Yu Chu[1], Qiyang Chen[3], Andrew Breon[4], Sibo Li[5], Matthew B. Wielgat[7], Dhanansayan Shanmuganayagam[7,8,9], Edith Tzeng[10], Xuecang Geng[4], Kang Kim[2, 3, †], Xiaoning Jiang[1, †]

[*] These authors equally contributed to this work

[†] Corresponding author. Email: kangkim@upmc.edu, xjiang5@ncsu.edu

[1]Department of Mechanical and Aerospace Engineering, North Carolina State University, Raleigh, NC, USA

[2]Department of Medicine, University of Pittsburgh, Pittsburgh, PA, USA

[3]Department of Bioengineering, University of Pittsburgh, Pittsburgh, PA, USA

[4]Blatek Inc., Boalsburg, PA, USA

[5]Shenqi Medical (USA) Sirius Technologies Ltd., Boston, MA, USA

[6]Department of Mechanical Engineering, Inha University, Incheon, South Korea

[7]Department of Animal and Dairy Sciences, University of Wisconsin-Madison, Madison, WI, USA

[8]Department of Surgery, University of Wisconsin School of Medicine and Public Health, Madison, WI, USA

[9]Center for Biomedical Swine Research & Innovation, University of Wisconsin-Madison, Madison, WI, USA

[10]Department of Surgery, University of Pittsburgh, Pittsburgh, PA, USA

This PDF file includes:

- supplementary tables S1 to S5
- supplementary figures S1 to S5

TABLE S1. SUMMARY OF HEATING ULTRASOUND TRANSDUCER IN CURRENT US-TSI DEVICES.

Author	Year	Test	Target	Transducer	Frequency*	Intensity†	Temperature rise	Heating duration	Heating volume**	References
Huang et al	2007	*In vitro*	Phantom	32-element array	6.7 MHz	320 W/cm^2 I_{SPPA}	1.0 °C	1.1 s	N/A	[30]
Liang et al	2008	*In vitro*	Phantom	128-element, linear array (Ultrasonix L14-5W/60)	5.6 MHz	195 W/cm^2 I_{SPPA}	1.7 °C	2000 s	N/A	[56]
Kim et al	2008	*In vitro*	Porcine artery	513-element, 2D phased array	1.0 MHz	2000 W/cm^2 I_P	3.2 °C	2.0 s	5 x 5 x 10 mm^3	[34]
Stephens et al	2013	*In vitro*	Phantom	6 single-element, flat or spherical aperture	3.5 MHz<br>4.0 MHz	56 W/cm^2 I_P<br>40 W/cm^2 I_P	3.0 °C	2.0 s	2 x 8 x 5 mm^3	[35]
Mahmoud et al	2013	*In vivo*	New Zealand white rabbits	Custom array	3.55 MHz	N/A	1.1 ± 0.1 °C	5.0 s	N/A	[31]
Mahmoud et al	2014	*Ex vivo*	Mouse livers	6 single-element	3.55 MHz	117 W/cm^2 I_{SPPA}	1.5 °C	3.0 s	N/A	[57]
Foiret et al	2015	*In vitro*	Phantom	1 single-element (Valpey-Fischer IL0508HP)	5.0 MHz	N/A	3.8 °C	30 s	0.9 x 0.9 mm^2	[58]
Nguyen et al	2017	*In vitro*	Phantom	Linear array (Philips ATL L7-4)	5.0 MHz	1040 W/cm^2 I_P	0.3 °C	2.0 s	8.5 x 10 mm^2	[36]
Khalid et al	2021	*In vitro*	Phantom	Curved linear array (Philips ATL C4-2)	3.0 MHz	354 W/cm^2 I_{SPPA}	1.0 °C	2.0 s	14 x 64 mm^2	[37]
Khalid et al	2021	*In vitro*	Phantom	Custom array	3.55 MHz	66 W/cm^2 I_{SPPA}	1.5 °C	3.0 s	8 x 5 mm^2	[59]
Our work‡	2024	*In vitro*<br>*In vivo*	Phantom<br>Pig	32-element, dual 1D concave arrays	3.5 MHz	1300 W/cm^2 I_{SPPA}	3.9 °C<br>2.0 °C	25 ms<br>50 ms	2 x 10 x10 mm^3	N/A

* I_{SPPA} refers to spatial peak pulse average intensity, and I_P refers to spatial peak intensity.

† Heating volume was represented in elevation× lateral x depth directions or lateral x depth directions.

‡ For *in vitro* tests, a 3.9 °C temperature rise was obtained within 25 ms of heating in phantom; for *in vivo* tests, a 2.0 °C temperature rise was obtained within 50 ms of heating in pig.

TABLE S2 MATERIAL PROPERTIES OF FINITE ELEMENT SIMULATION

Properties	PZT-5A	Properties	Al_2O_3/Epoxy	Graphite
Density	7750 kg/m^3	Density	2700 kg/m^3	2260 kg/m^3
C_{33}^{E}	111 GPa	Young's modulus	11.5 GPa	36.5 GPa
e_{33}	15.8 C/m^2	Poisson's ratio	0.32	0.19

TABLE S3 PERFORMANCE COMPARISONS OF HEATING TRANSDUCER ELEMENTS

	Material: PZT 5A	Material: PZT 5H	Material: PMN-PT
Impedance analysis	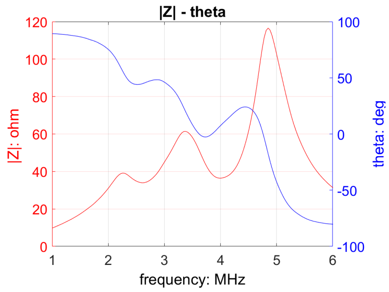 Impedance (@ 3.5 MHz): 58 Ω<br>Phase angle (@ 3.5 MHz): 8.6°	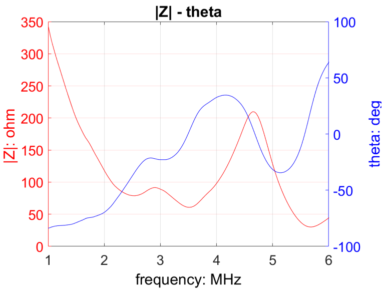 Impedance (@ 3.5 MHz): 62 Ω<br>Phase angle (@ 3.5 MHz): 0.9°	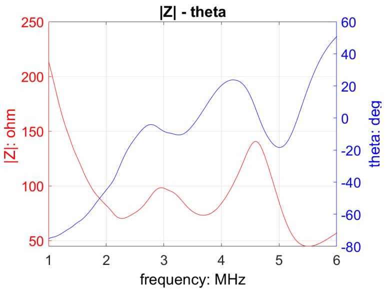 Impedance (@ 3.5 MHz): 78 Ω<br>Phase angle (@ 3.5 MHz): -4.9°
Pulse echo test	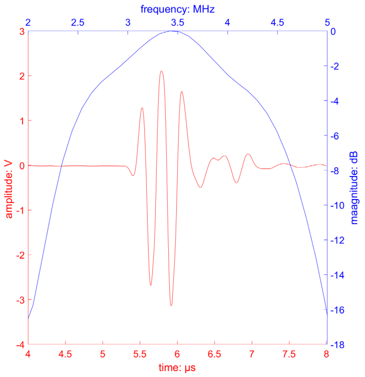 Center frequency: 3.47 MHz<br>Bandwidth (-6 dB): 60.3%<br>Loop sensitivity: -15.6 dB	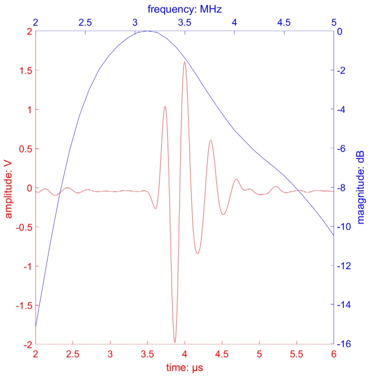 Center frequency: 3.27 MHz<br>Bandwidth (-6 dB): 56.0%<br>Loop sensitivity: -18.9 dB	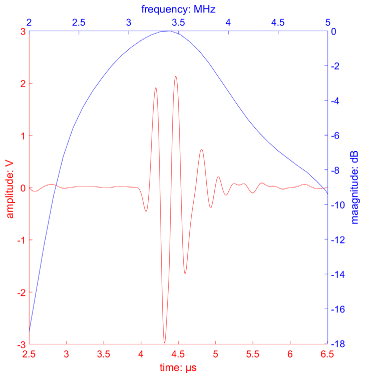 Center frequency: 3.37 MHz<br>Bandwidth (-6 dB): 57.0%<br>Loop sensitivity: -15.8 dB
Hydrophone test	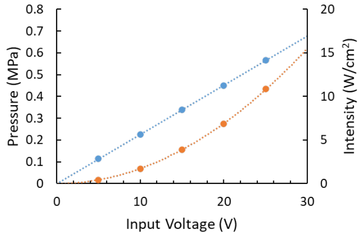 Transmitting sensitivity<br>(@ 5 cycles): 22.67 kPa/V	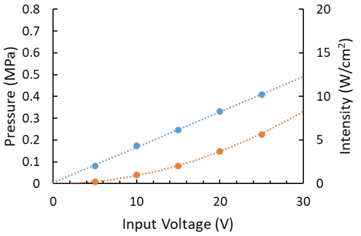 Transmitting sensitivity<br>(@ 5 cycles): 16.34 kPa/V	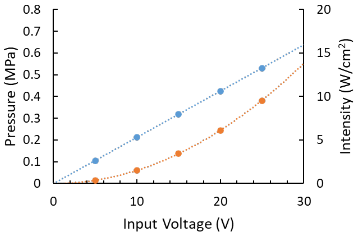 Transmitting sensitivity<br>(@ 5 cycles): 21.23 kPa/V

TABLE S4 SPECIFICATION OF UTILIZED THERMOCOUPLES

Item	Thermocouple No. 1	Thermocouple No. 2
Model	Ultra-fast response bare foil thermocouple	CO_2-T
Manufacturer	RdF Corporation, NH, USA	Omega Engineering, Inc., CT, USA
Response time	1 - 5 ms	2 - 5 ms
Foil Thickness	12.7 μm (0.0005”)	12.7 μm (0.0005”)
Temperature range	-195.56 - 371.11°C	< 150.00°C
Total length	150 mm (6”)	150 mm (6”)

TABLE S5 ELECTRICAL IMPEDANCE SPECTRUM OF EACH HEATING ELEMENT

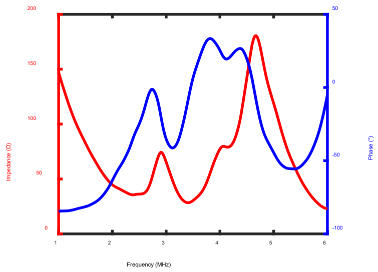

No. 1

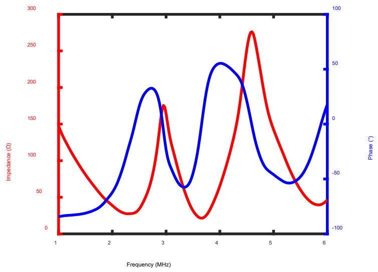

No. 2

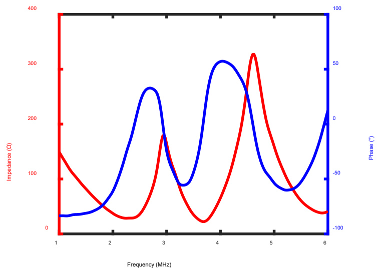

No. 3

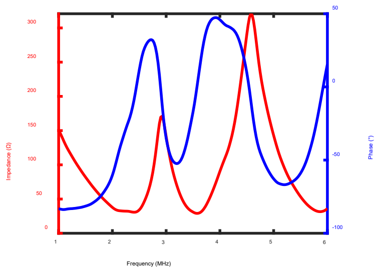

No. 4

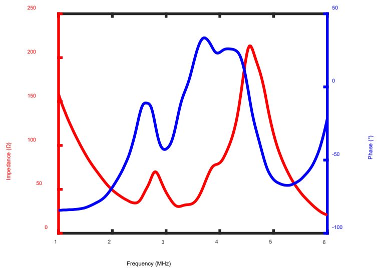

No. 5

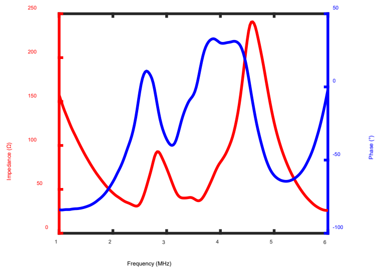

No. 6

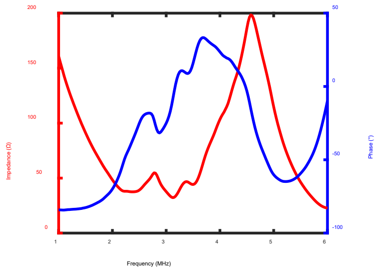

No. 7

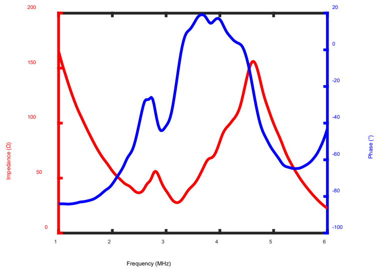

No. 8

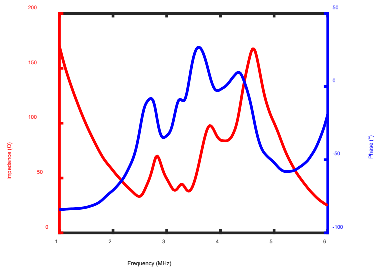

No. 9

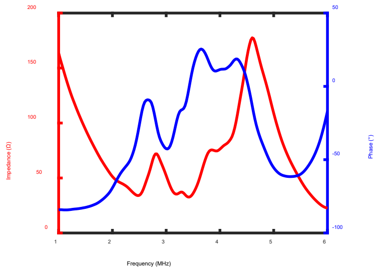

No. 10

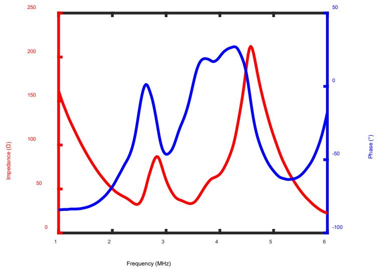

No. 11

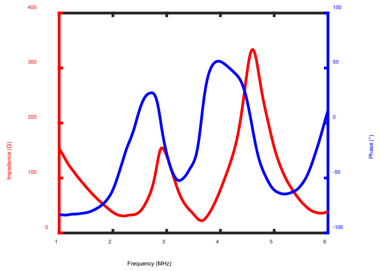

No. 12

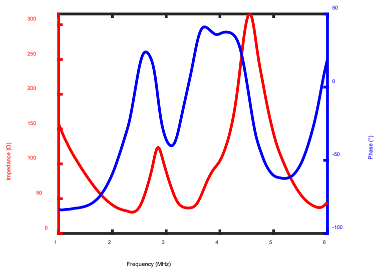

No. 13

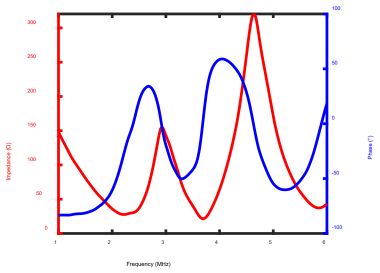

No. 14

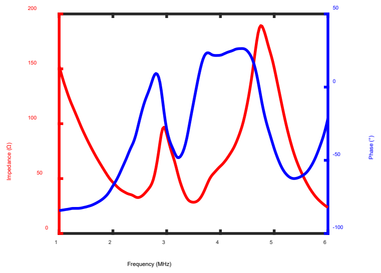

No. 15

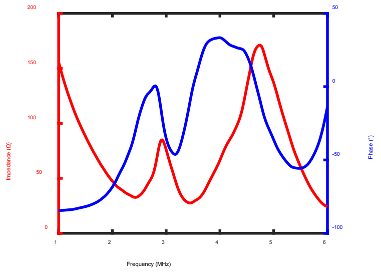

No. 16

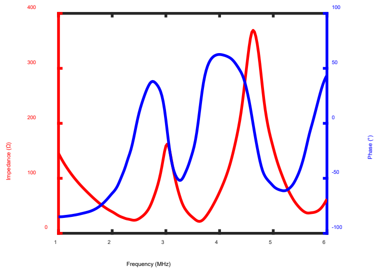

No. 17

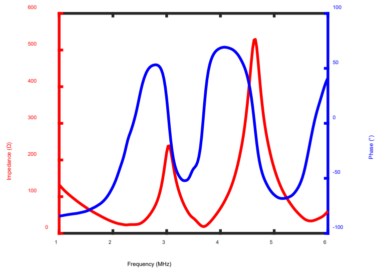

No. 18

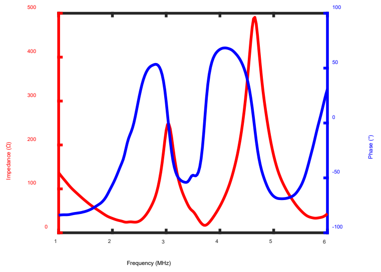

No. 19

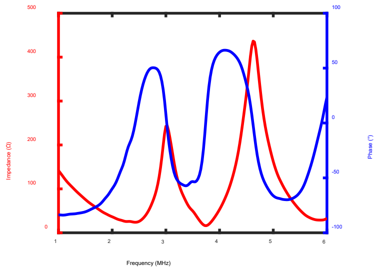

No. 20

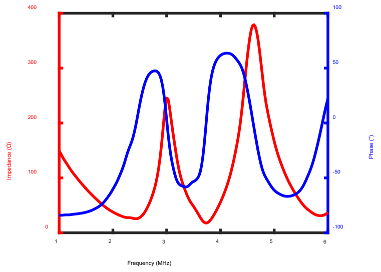

No. 21

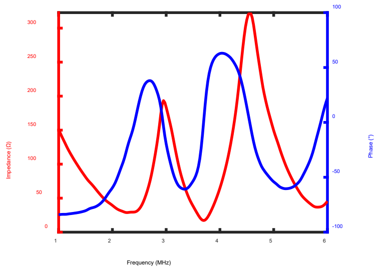

No. 22

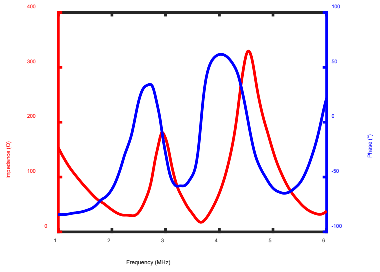

No. 23

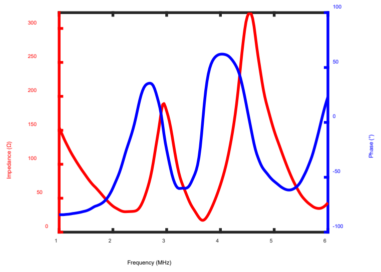

No. 24

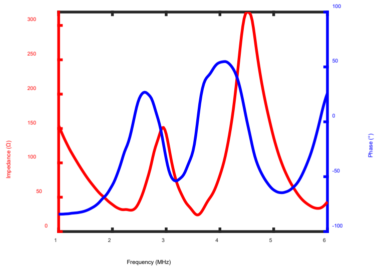

No. 25

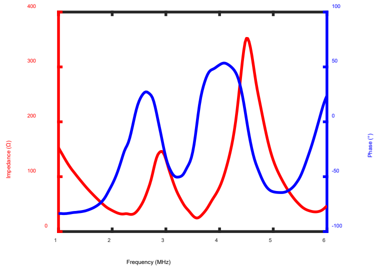

No. 26

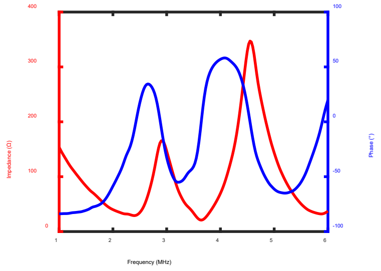

No. 27

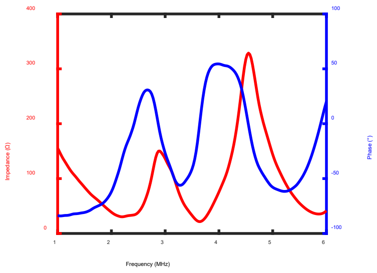

No. 28

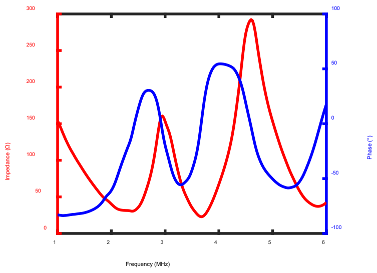

No. 29

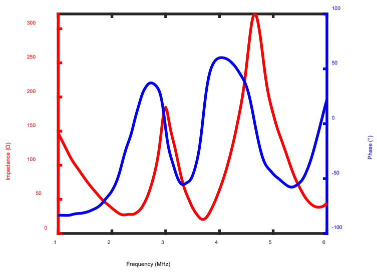

No. 30

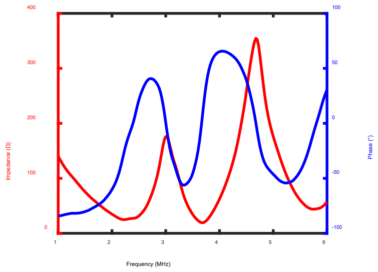

No. 31

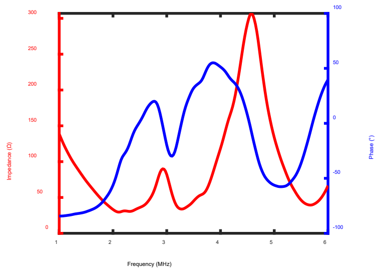

No. 32

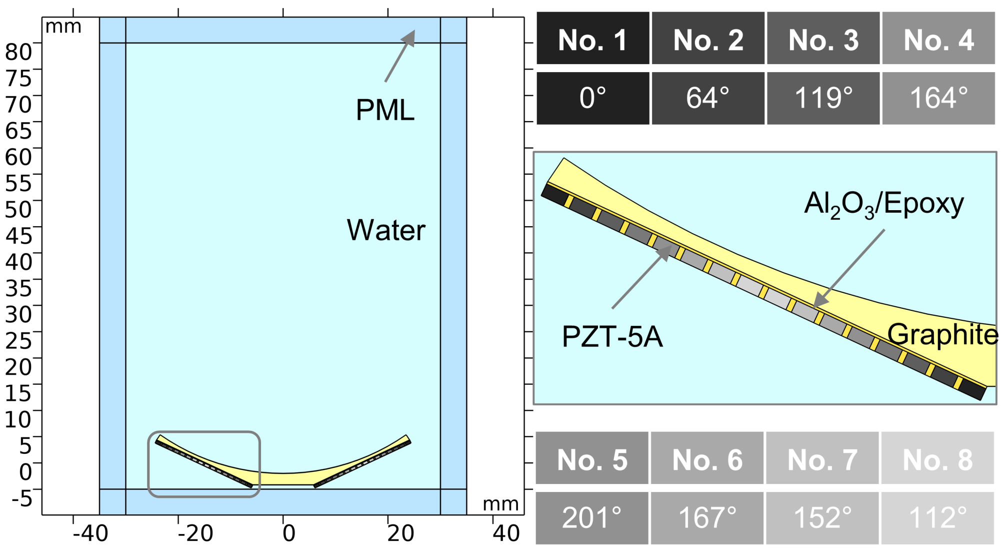

Figure S1. Acoustic simulation modeling for the heating transducer, with multi-focus beamforming applied by programming phase delay on each heating element.

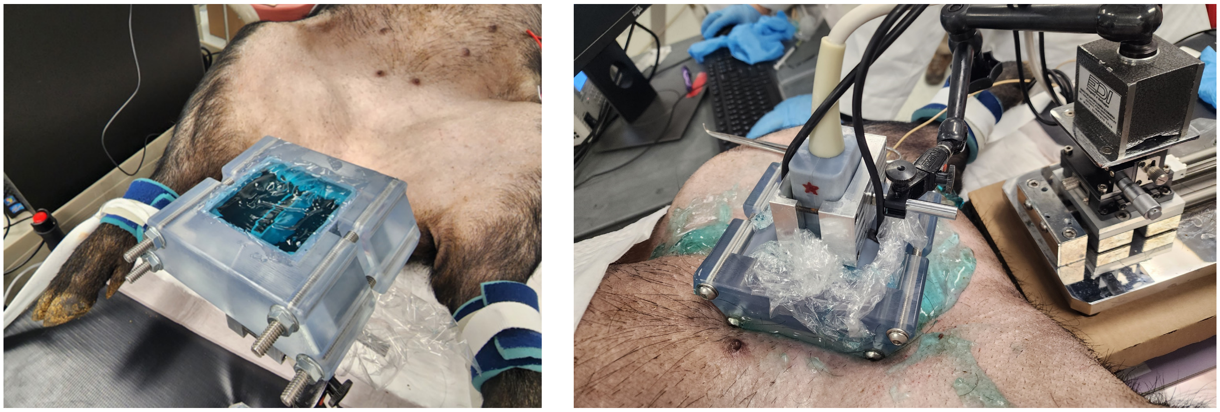

A B

Figure S2. Photo of experiment setup for *in vivo* animal tests.

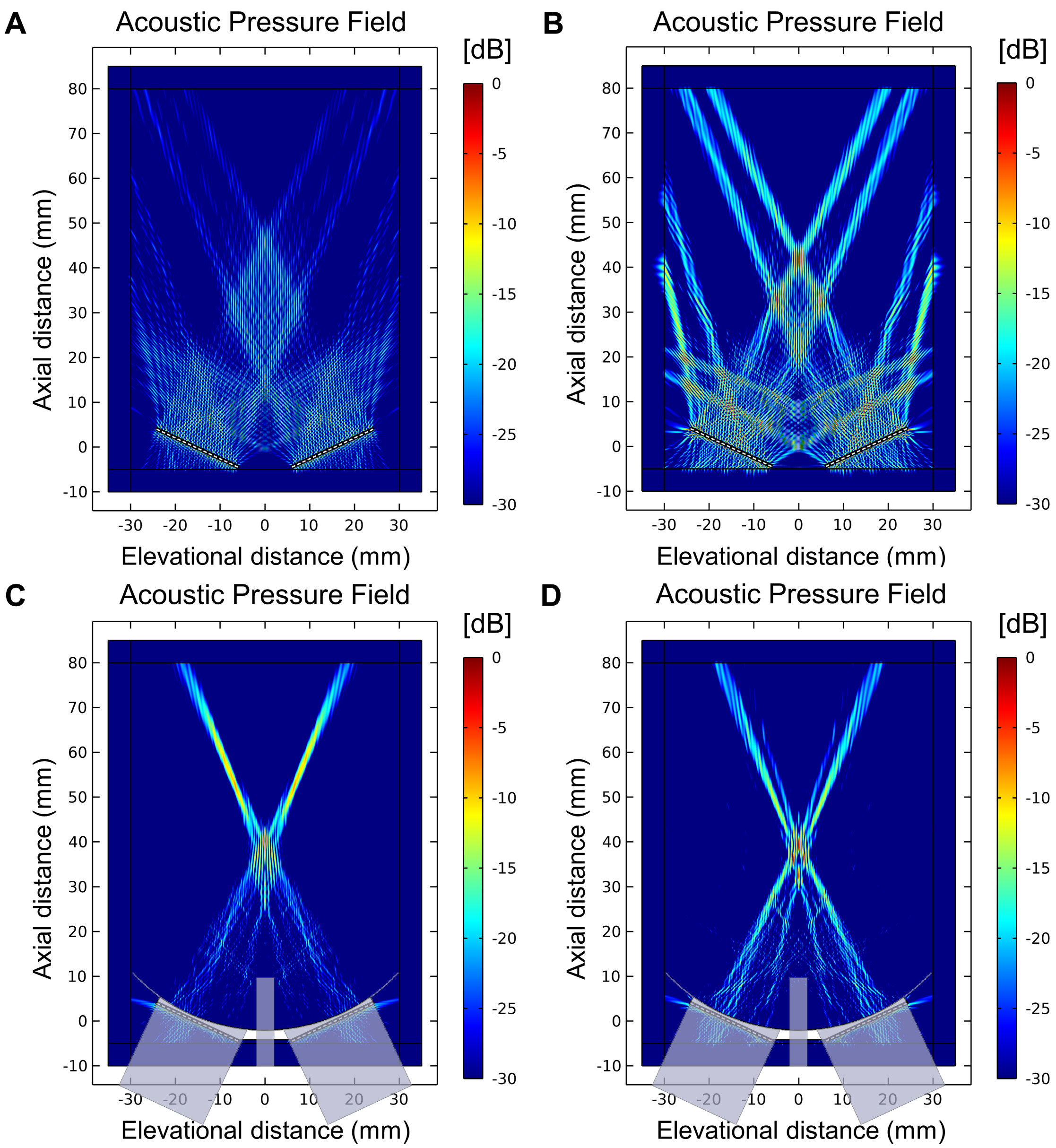

Figure S3. Simulated acoustic pressure field of the designed heating transducer in the YZ plane, including (A) not applying acoustic lens neither phase delay; (B) applying phase delay but not acoustic lens; (C) applying acoustic lens but not phase delay; (D) applying both acoustic lens and phase delay.

The absence of phase delay and acoustic lens clearly resulted in broad ultrasound beams (Figure S3A) lacking the beam focusing necessary to elevate acoustic pressure. The desired multi-focus beamforming could be achieved once the phase delay was applied to each heating element, as depicted in Figure S3B. Nevertheless, the lack of an acoustic lens contributed to a wide region of low acoustic pressure between each focal point. When only the acoustic lens was applied to the heating transducer, the simulated acoustic pressure field (Figure S3C) showed that there is a relatively high-pressure area generated at a focal depth of approximately 35 mm. However, two higher acoustic pressure regions were observed behind the intended focal area due to the absence of the necessary phase delay. When both acoustic lens and phase delay were applied, the simulated results (Figure S3D) demonstrated that the focal area of a single heating array overlapped with the other very well. The generated focal area of dual heating arrays had an approximate -12 dB beamwidth of 10 mm.

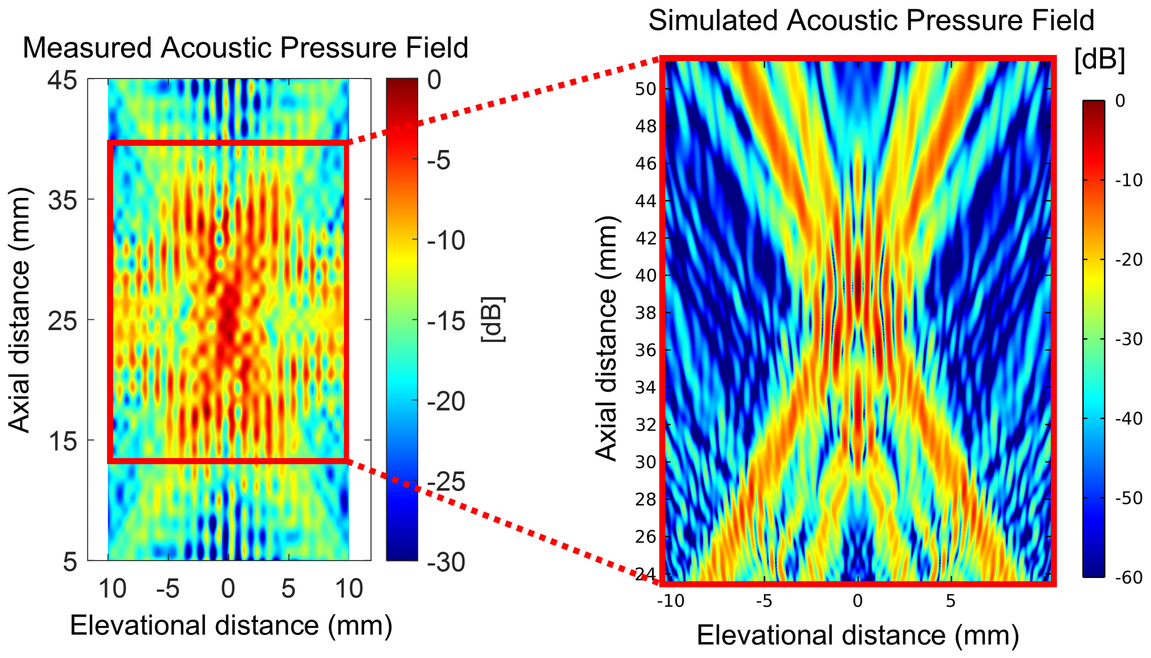

Figure S4. Comparison between measured and simulated acoustic pressure field of the designed heating transducer in YZ plane. Note that the coordinate systems for the simulated and measured acoustic pressure maps differ. In the measured map, the focal spot is located at an axial distance of 25 mm, whereas in the simulated map, it is at 35 mm.

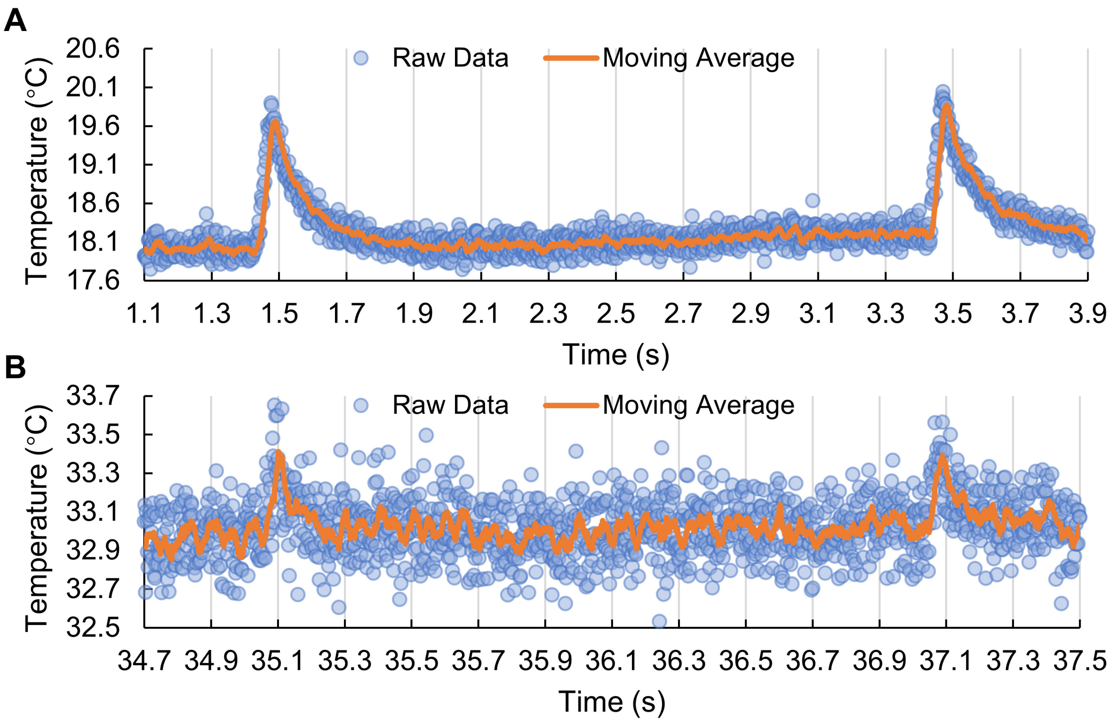

Figure S5. Measured transient temperature curve using fast-response thermocouple in (A) laser-induced thermal tests and (B) ultrasound-induced thermal tests.

References

56. Liang H-D, Zhou L-X, Wells PNT, Halliwell M. Temperature Measurement by Thermal Strain Imaging with Diagnostic Power Ultrasound, with Potential for Thermal Index Determination. Ultrasound Med Biol. 2009; 35: 773–80.

57. Mahmoud AM, Ding X, Dutta D, Singh VP, Kim K. Detecting hepatic steatosis using ultrasound-induced thermal strain imaging: An ex vivo animal study. Phys Med Biol. 2014; 59: 881–95.

58. Foiret J, Ferrara KW. Spatial and Temporal Control of Hyperthermia Using Real Time Ultrasonic Thermal Strain Imaging with Motion Compensation, Phantom Study. Talkachova A, Ed. PLoS One. 2015; 10: e0134938.

59. Khalid WB, Farhat N, Lavery L, Jarnagin J, Delany JP, Kim K. Non-invasive Assessment of Liver Fat in ob/ob Mice Using Ultrasound-Induced Thermal Strain Imaging and Its Correlation with Hepatic Triglyceride Content. Ultrasound Med Biol. 2021; 47: 1067–76.
